# Protein Requirements for Master Athletes: Just Older Versions of Their Younger Selves

**DOI:** 10.1007/s40279-021-01510-0

**Published:** 2021-09-13

**Authors:** Daniel R. Moore

**Affiliations:** Faculty of Kinesiology and Physical Education, University of Toronto, 100 Devonshire Place, Toronto, ON M5S 2C9 Canada

## Abstract

It is established that protein requirements are elevated in athletes to support their training and post-exercise recovery and adaptation, especially within skeletal muscle. However, research on the requirements for this macronutrient has been performed almost exclusively in younger athletes, which may complicate their translation to the growing population of Master athletes (i.e. > 35 years old). In contrast to older (> 65 years) untrained adults who typically demonstrate anabolic resistance to dietary protein as a primary mediator of the ‘normal’ age-related loss of muscle mass and strength, Master athletes are generally considered successful models of aging as evidenced by possessing similar body composition, muscle mass, and aerobic fitness as untrained adults more than half their age. The primary physiology changes considered to underpin the anabolic resistance of aging are precipitated or exacerbated by physical inactivity, which has led to higher protein recommendations to stimulate muscle protein synthesis in older untrained compared to younger untrained adults. This review puts forth the argument that Master athletes have similar muscle characteristics, physiological responses to exercise, and protein metabolism as young athletes and, therefore, are unlikely to have protein requirements that are different from their young contemporaries. Recommendations for protein amount, type, and pattern will be discussed for Master athletes to enhance their recovery from and adaptation to resistance and endurance training.

## Key Points

**Table:** 

Master athletes are models of successful aging and are not susceptible to ‘normal’ age-related muscle loss or ‘anabolic resistance’ to dietary protein.
Current protein recommendations for resistance and endurance trained younger adults are applicable to Master athletes.

## Introduction

Dietary protein supports athlete’s training and adaptation by providing the essential amino acid building blocks for the remodeling of muscle and body proteins. While carbohydrate and fat represent the primary energy sources during exercise, amino acid oxidation may contribute up to 10% of energy during exercise when carbohydrate availability is low [1, 2]. Thus, research has clearly established that daily protein requirements are elevated above the current minimum daily recommended allowance of ~ 0.8 g/kg/day across the spectrum of sport disciplines [3]. However, the majority of research that has contributed to these increased recommendations has been performed in active young athletes (18–35 years). The aging of these younger athletes and a greater awareness of an active lifestyle to support successful aging has contributed to expansion of athletes above the age of 35 years [4], which is the typical delineation for Master athlete although this may be discipline-specific. Therefore, there is a need to clearly define the nutrient requirements of these older athletes to support their training, adaptation, and health.

Muscle mass and strength as well as aerobic capacity generally peak in early adulthood (< 30 years) after which they begin a slow, insipid decline (~ 0.5–1%/year) that can accelerate in the 8th decade [5, 6]. However, high levels of physical activity typical of Master athletes tend to spare older adults from the typical loss of muscle and fitness that besieges their sedentary peers [7]. For example, a comprehensive review and meta-analysis by McKendry et al. [8] demonstrated that Master athletes’ aerobic capacity, muscle strength, and body composition are generally on par with young, untrained adults. The benefits of chronic endurance training are not only afforded to life-long athletes as late starting (i.e. > 50 years) Master athletes have similar aerobic capacity and muscle quality as those who maintained training from early adulthood [9]. Thus, while there is an inevitable decline in exercise capacity and performance with age, Master athletes are generally regarded as models of successful aging [7, 10]. Therefore, the question of whether other sequelae of aging that may impact protein requirements are observed in Master athletes warrants discussion. The present review will summarize the available evidence on the primary factors associated with typical age-related anabolic resistance and discuss to what extent they may be present in Master athletes. In addition, although there is relatively little research in middle-aged adults, who nonetheless could still qualify categorically as Master athletes, the review will primarily focus on older (i.e. > 60 years) adults and athletes to reflect the majority of research in this discipline. This older demographic (i.e. > 60 years) is also the primary focus of research that explores the potentially greater meal protein requirements for skeletal muscle remodeling in non-exercising older as compared to young adults (e.g. [11]). Therefore, the perception that protein requirements may differ in Master athletes is arguably more relevant to these older as compared to middle-aged Master athletes due to their greater chronological age. However, middle-aged Master athletes adhering to the recommendations discussed below and largely established in younger athletes would satisfy their protein requirements as well. Finally, the review will discuss daily protein recommendations determined using (traditional) whole body methodology (e.g. nitrogen balance, stable isotopes) but will also highlight what meal protein intake would optimize muscle protein remodeling; this latter approach will leverage knowledge from contemporary stable isotope techniques and has been used to define optimal daily protein intakes when suggesting optimal meal frequencies in older untrained adults (e.g. [12]). Thus, given meal frequencies are generally greater in athletes than untrained individuals, providing recommendations based on meal protein targets can also influence daily protein intakes, as will be discussed in more detail below.

## Anabolic Resistance of Aging

**Fig. 1:**
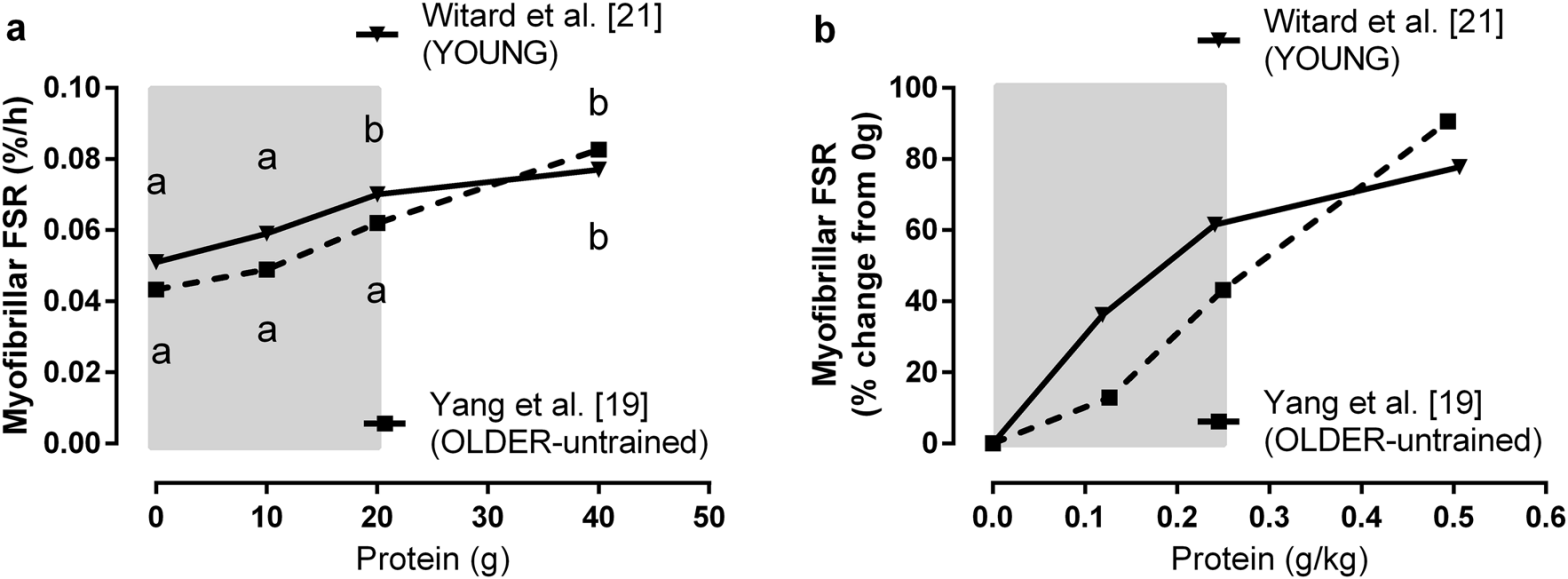
**a** Myofibrillar protein synthesis after an acute bout of resistance exercise and graded intakes of whey protein in young trained [21] and older untrained [19] adults. Doses with different letters are significantly from each other within a population. **b** Data from these same publications [19, 21] expressed as the increase in myofibrillar protein synthesis after resistance exercise from a protein-free control against the body weight-normalized whey protein intake. Shaded area highlights the relative anabolic resistance of the untrained older adults. *FSR* fractional synthesis rate

The age-related decrements in muscle size and function are multifactorial and could include decreases in neuromuscular function, motor unit/fibre loss (especially of the type II fibres), muscle stem cell dysregulation, and/or atrophy of skeletal muscle, the latter of which is largely underpinned by an “anabolic resistance” to dietary protein and will be the primary focus of the present review (for reviews on the other factors, see [13–15]). This anabolic resistance is characterized by a blunted muscle protein synthetic response to bolus (i.e. meal) dietary amino acid ingestion at rest [16–18] and after resistance exercise in older untrained adults [19, 20] and is most pronounced in the myofibrillar protein fraction, which is critical for the maintenance of muscle mass and function given it represents the predominate fraction (~ 60% of total muscle protein) and includes the contractile elements of the sarcomere (e.g. actin, myosin). For example, an estimated average meal protein requirement of ~ 0.4 g/kg of high-quality, leucine-enriched protein (i.e. whey) is required to maximize myofibrillar protein synthesis (MyoPS) at rest in untrained older adults, which is ~ 65% greater than for younger adults [17]. Similarly, 20 g of high-quality protein (i.e. whey) is sufficient to maximally increase MyoPS after exercise in young, trained men, whereas older, untrained adults require up to 40 g [19, 21], findings that are also evident when the protein intakes are normalized to body mass (Fig. 1). Importantly, older untrained muscle is generally able to mount an equivalent MyoPS response to that of young adults when meal protein ingestion is at or above its respective maximal threshold [17, 19, 21], although this finding is not without exceptions [16]. Thus, this anabolic resistance to suboptimal meal protein intakes has contributed to the recommendations that older untrained adults require a greater meal protein intake to maximize muscle protein synthesis at rest [12] and after resistance exercise [11]. However, indiscriminately consuming supramaximal meal protein intakes can be inefficient for optimizing muscle remodeling as excess dietary protein may be retained in nonmuscle tissue [22] or be diverted to amino acid oxidation [19, 21, 23]. Therefore, subsequent meal protein targets will be discussed in relation to optimizing the efficiency of dietary amino acid ingestion insofar as maximizing muscle protein synthesis yet minimizing amino acid oxidative losses.

Anabolic resistance to dietary amino acids is multifactorial and could include chronic inflammation, reduced capillarity and/or vasodilatory capacity (and hence amino acid delivery), attenuated muscle uptake (e.g. amino acid transporter expression/function), and/or reduced ribosomal protein content or activity. However, Master athletes are regarded as successful models of aging [10] and are distinguished from their nonactive peers, on which the majority of aging research is focused, by their high levels of habitual activity. Thus, it is questionable whether meal protein recommendations for older untrained adults, which typically advocate for larger protein ingestions to offset any age-related anabolic resistance [11], are relevant for Master athletes. In light of the lack of specific research investigating daily or, especially, meal protein requirements of older trained adults, the following sections will discuss whether some of the factors commonly associated with or that underpin anabolic resistance could be present in Master athletes.

### Digestion and Absorption

Aging has been shown to reduce the appearance of dietary amino acids in the systemic circulation during the postprandial period [24, 25], which could reduce their availability for uptake by skeletal muscle. By first principles, this reduced amino acid appearance could include reduced digestion/absorption and/or increased first pass splanchnic extraction. There is evidence that whole foods that require effective mastication prior to their digestion are associated with a reduced or delayed postprandial aminoacidemia in older adults, especially those with dentures [26, 27]. Although data are not available in Master athletes, young athletes have been reported to have a relatively high incidence of dental issues (e.g. caries, dental erosion, etc.), which may be related in part to dietary habits, such as high carbohydrate intakes (especially simple sugars), required to sustain optimal training and performance [28]. If these trends persist with advanced training, then it is possible they may compound the typical age-related declines in dental health, which could predispose Master athletes to reduced amino acid availability secondary to reduced chewing efficiency. Notwithstanding differences in whole food digestion, aging per se has been associated with a greater splanchnic extraction of dietary amino acids even from liquid protein sources (e.g. milk and dairy-based beverages), although this difference is relatively minor amounting to only ~ 6% of an ingested dose [25]. Of potential relevance to athletes is that exercise in untrained young adults can reduce the rate of appearance of amino acids in the circulation after protein beverage ingestion, which was suggested to be related to ischemia/reperfusion intestinal damage [29, 30]. However, despite similar increases in estimates of intestinal damage (i.e. circulating intestinal fatty acid binding protein; iFABP) after moderate-intensity exercise in trained young adults there is no impact on the appearance of dietary protein-derived amino acids when consumed within a mixed macronutrient meal during the early postprandial period [31]. This could suggest that athletes who regularly expose their splanchnic region to exercise-induced ischemia–reperfusion have conditioned these tissues so that any post-exercise remodeling, as suggested by circulating intestinal cell markers (i.e. iFABP), does not compromise cellular function. Thus, provided dental health is maintained, there is insufficient evidence to suggest that Master athletes would suffer from age-related anabolic resistance secondary to increased splanchnic amino acid sequestration that may affect their sedentary counterparts.

### Amino Acid Delivery

The delivery of dietary amino acids to skeletal muscle is mediated by an extensive network of capillaries and their ability to undergo insulin-induced dilation during the postprandial period. Older untrained adults exhibit both a reduction in the capillary:fibre ratio [32] and an attenuated insulin-induced capillary vasodilation [33], the latter of which is associated with a blunted muscle protein synthetic response to exogenous amino acids [34, 35]. However, the apparent age-related decline in muscle capillarity may be largely influenced by habitual activity as training can induce angiogenesis in older adults [36, 37] whereas inactivity may induce rarefaction [38, 39]. Moreover, insulin-induced nutritive blood flow (i.e. microvascular perfusion) can be improved in older adults for up to 16 h by as little as 45 min of moderate-paced walking, which is concomitant with an increase in intramuscular anabolic signaling (e.g. mechanistic target of rapamycin; mTOR) and muscle protein synthesis to exogenous insulin and/or amino acids [40, 41]. Collectively, these data highlight the important role for habitual activity in capillarity and microvascular responsiveness to dietary nutrients. Importantly, intravenous administration of insulin and amino acids increases microvascular blood flow in trained but not untrained older adults, which is accompanied by a moderately greater (but not statistically different) increase in MyoPS (estimated effect size: 0.50; 95% CI − 0.41 to 1.37)[42]. Thus, given Master athletes would have training volumes that exceed their untrained peers and possess capillary networks that are on par with their younger contemporaries [43], it is unlikely that nutrient (insulin and amino acid) delivery to skeletal muscle is impaired in these athletes.

### Amino Acid Uptake

Amino acid uptake into skeletal muscle has been suggested to represent a locus of control for skeletal muscle anabolism that may be compromised in older muscle [44]. During the immediate post-exercise period, there is an increase in the inward transport of amino acids that is enhanced in the presence of insulin and exogenous amino acids in young adults [45–47]. The uptake of amino acids is facilitated by transmembrane proteins that may also act as “transceptors” as a feedforward mechanisms to enhance mTORC1 activation [48]. The potential role for these transporters in any age-related anabolic resistance is unclear given that studies to date have largely been limited to assessing their gene or protein expression but not their intracellular localization or activity. Some research suggests that protein expression is differentially regulated by age, resistance exercise, and amino acid ingestion (for review, see [44]). For example, acute resistance exercise has been reported to increase protein expression of the sodium-coupled neutral amino acid transporter 2 (SNAT2) and the L-type amino acid transporter 1 (LAT1) in older untrained adults [49, 50] with general trends to an increased expression in a muscle membrane-enriched fraction [51]. The potential implication of these results is that SNAT2 and LAT1 work in concert to increase cellular uptake of the anabolic essential amino acid leucine [52]. Thus, a greater expression (and potentially at the muscle membrane) of these transporters could improve the ability to increase the capacity for amino acid uptake and would be concomitant with previously reported increased free-living rates of MyoPS with exercise and protein ingestion in older untrained adults [53]. Recent research in young adults also suggests that resistance training can increase the plasma membrane expression of LAT1 [54]. However, much is not known about the biological relevance of increased amino acid transporter expression given this is bi-directional in the transmembrane exchange of amino acids and, therefore, may just represent an increased capacity for muscle amino acid flux. Nevertheless, while studies in Master athletes are lacking, the observation that inactivity can reduce amino acid transporter expression [55], whereas activity can increase its expression in young and older adults could collectively suggest the presence of sufficient amino acid transporters would not represent a rate-controlling step for post-exercise anabolism in concurrently training Master athletes.

### mRNA Translation

The blunted MyoPS response to exercise or exogenous amino acids in older untrained adults has been suggested to be related to an inability to activate (as suggested by protein phosphorylation) components of the mTORC1 signaling pathway [16, 56, 57] but not to inadequate translational machinery (i.e. ribosomal protein), the latter of which has been reported to be paradoxically greater in older skeletal muscle [58]. However, as highlighted previously, even moderate exercise (i.e. 45 min of walking) can increase the ability of dietary amino acids to activate this anabolic pathway and increase postprandial protein synthesis in older untrained men [41]; this highlights the important role for prior muscle contraction to sensitize this signaling pathway in older adults, which can also occur in younger individuals for up to 24 h after resistance exercise [59].

Research is also revealing the dynamic regulation of mTORC1 in human muscle through its intracellular translocation and association with positive regulators [60]. For example, translocation of mTORC1 to the lysosomal membrane is integral for its downstream kinase activity and MyoPS during recovery from exercise and protein ingestion in young adults [61–63]. While there is limited research in older human muscle, it is revealing that mTOR-lysosomal colocalization after essential amino acid ingestion is increased after 12 weeks of training in older muscle, which was associated with ~ 20% greater postprandial mixed muscle protein synthetic response [64]. Thus, it appears that conditioned older muscle retains the ability for intracellular mTORC1 translocation that is required for its activation.

### Inflammation

Aging may be associated with a chronic subclinical systemic and intramuscular inflammation, which is thought to contribute to the anabolic resistance of aging [16]. However, this inflammatory status may be underpinned or exacerbated by inactivity and/or obesity [65–67], highlighting potential modifiable risk factors for its etiology. For example, resistance training has been shown to reduce the expression of tumor necrosis factor-α in the muscle of frail older adults [68]. Moreover, combined aerobic and resistance exercise, but not weight loss alone, decreases the intramuscular expression of inflammatory markers in frail obese older adults [69]. It is perhaps, therefore, unsurprising that Master athletes have similar resting levels of inflammatory markers as young adults [70] and experience attenuated exercise-induced increases in some of these markers compared to untrained older adults, although this effect may be more pronounced in males as compared to females [71, 72]. Therefore, chronic intramuscular inflammation (which may be linked to anabolic resistance) is unlikely to be present in Master athletes, although in keeping with sports science research in general in young adults [73], additional research is warranted in females.

### Is There Evidence for Reduced Post-exercise Anabolic Resistance in Active Older Adults?

**Fig. 2:**
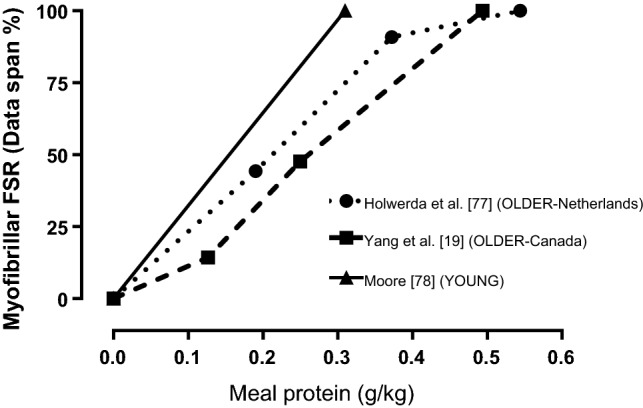
Myofibrillar protein synthesis after resistance exercise in response to dietary protein ingestion in young adults [78] and untrained older adults in different geographical locations who may have relatively higher [77] or lower [19] habitual activity levels. Data are normalized to the maximal myofibrillar protein synthetic response within each study (i.e. data span) assuming a maximal response at the highest intake (40–45 g of protein or > 0.49 g/kg). Error bars have not been included due to the requirement to normalize intakes and percentage of maximal response to reported mean group values from the respective publications. *FSR* fractional synthesis rate

Studies investigating the post-exercise ingested bolus protein dose–response in Master athletes do not currently exist. However, some evidence for a reduced anabolic resistance after exercise may be drawn from populations of older adults who presumably have different levels of habitual physical activity or reside in communities that may be more conducive to greater levels of non-exercise adaptive thermogenesis (e.g. walking or cycling for transport). For example, the Netherlands is on average one of the most active countries in Europe [74] with older adults’ (61–70 years) reported daily walking and cycling activity generally exceeding ~ 2 h/day [75]. Canada, in contrast to many European countries, has a large activity variability (Gini coefficient) of the population which has been shown to align with rates of obesity and walkability of cities [76]. While studies do not consistently report on the baseline physical activity or fitness parameters of participants, it is notable that the post-exercise dose–response studies in older untrained men have been performed in Canada and the Netherlands and could suggest an influence of baseline metabolic health on the requirement. For example, Holwerda et al. [77] in the Netherlands and Yang et al. [19] in Canada performed absolute protein dose–response studies of the myofibrillar protein fraction after an acute bout of resistance exercise with dairy protein (milk protein concentrate and whey, respectively) ranging from 0 to 45 g in older untrained but otherwise healthy men. After normalizing the protein intake to body mass (similar to a previous report in young adults [78]) and adjusting MyoPS response to a data span (to account for methodological differences and lack of baseline control rates in both studies while assuming a similar maximal response with intakes of 40–45 g; Fig. 2), the relative dose–response curve was visually shifted to the left (i.e. greater sensitivity) in the older men from the Netherlands. While habitual activity of the study populations was not reported, it is notable that daily step counts in similar studies from McMaster University in Canada were ~ 6500 steps/day [66, 79], which is slightly below the recommended minimum target (7000 steps/day) to maintain physical health of older adults [80]. Therefore, although additional research would help confirm whether the thesis that habitual activity influences relative post-exercise protein requirements, these observations would be consistent with the ability of acute exercise to improve [40, 41] and reduced activity to impair [66, 67] the anabolic response to dietary amino acids in otherwise healthy older adults.

In more direct comparisons of the anabolic response of muscle protein synthesis to exogenous amino acids in trained older adults, it has been reported that basal and essential amino acid-induced stimulation of mixed muscle protein synthesis is ~ 20% greater after 12 weeks of resistance training in older adults [64]. In addition, a cross-sectional study reported that while basal and nutrient stimulated (i.e. intravenous glucose and amino acid infusion) rates of MyoPS are not statistically different between untrained and 20-week post-trained older adults, the increase from basal was numerically greater (i.e. ~ 75 vs. 43%) in the trained adults [42]. Thus, available evidence from acute dose–response studies, comparisons of trained and untrained older adults, and the general ‘young’ muscle phenotype of Master athletes would suggest they are unlikely to suffer from the typical age-related anabolic resistance, which incidentally is thought to be underpinned or exacerbated by physical inactivity [81, 82]. Therefore, the remainder of this review will discuss the current understanding of protein requirements in young adults as a reference point for older athletes.

## Resistance Exercise Protein Requirements

**Fig. 3:**
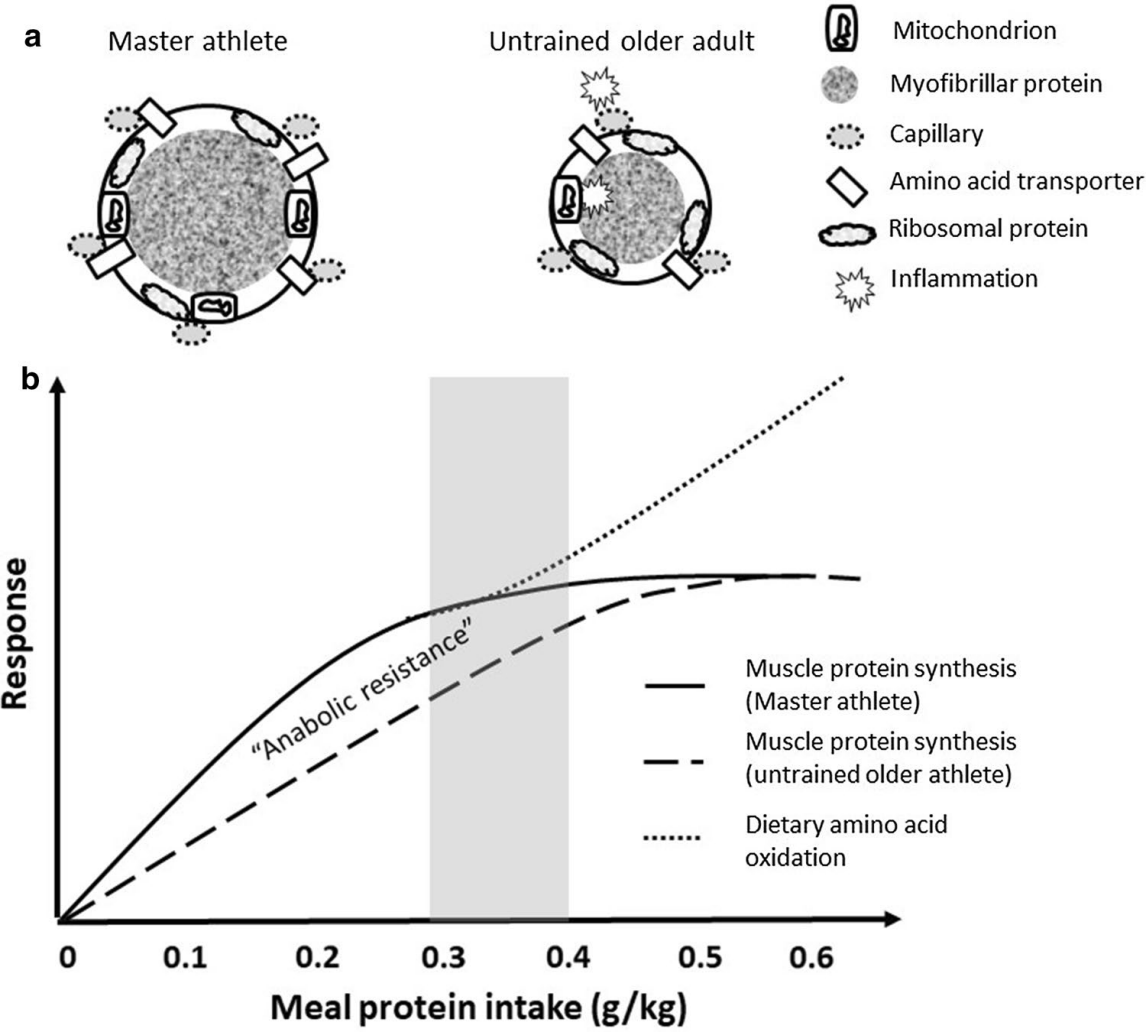
Schematic representation of the activity-related differences in muscle morphology **a** between Master athletes and older untrained adults that would influence the rested or post-exercise increase in muscle protein synthesis after meal protein ingestion (**b**). ‘Anabolic resistance’ is a rightward shift in the protein dose–response curve that is characteristic of an inactive lifestyle, especially with aging. Shaded area represents the meal protein intake that would maximize muscle protein synthesis yet minimize dietary amino acid oxidation in Master athletes

Resistance exercise is an inherently anabolic exercise modality that has been consistently shown to increase muscle protein turnover and attenuate the otherwise negative net muscle protein balance in the rested fasted state [45, 83]. However, consuming an exogenous source of amino acids is necessary to support maximal rates of muscle protein synthesis and a positive net protein balance, the latter of which is further supported by an ablation of the normal exercise-induced increase in muscle protein breakdown in the fasted state [46, 84, 85]. Current recommendations for young, resistance trained males is ~ 20 g of high-quality (i.e. leucine and essential amino acid-enriched) protein to maximize post-exercise rates of mixed muscle protein synthesis and MyoPS, while minimizing irreversible amino acid oxidative losses [21, 23]. Importantly, there is little evidence that young females exhibit a differential post-exercise muscle protein synthetic response with protein ingestion [86, 87], which permits a more generalizable recommendation of ~ 0.3 g/kg as an efficient target dose for resistance trained athletes [78]. Given the position above that there is little evidence for the presence of ‘anabolic resistance’ in Master athletes, this relative protein intake would represent a suitable target for older athletes as well to enhance muscle protein remodeling after a bout of resistance exercise (Fig. 3). However, should older athletes be concerned with the sufficiency of this intake then one could apply a safety margin for the estimated average requirement of 1.24, which represents the standard approach of twice the average assumed variance of 12% common to population level assessments [88]; this would, therefore, translate into ~ 0.37 g/kg or a modest ~ 28 g for a 75 kg athlete after exercise. While the impact of immediate post-exercise protein ingestion to enhance resistance training adaptations (e.g. strength, lean mass gains) has been reported to be small-to-negligible in mostly untrained individuals [89], it is beyond question that exogenous amino acid ingestion is required to maximize muscle protein synthesis [19, 21, 23, 77] and induce a positive muscle net protein balance [46, 84]; these outcomes would be consistent with the prudent advice that athletes consume a source of protein during the early post-exercise recovery period to facilitate recovery [3, 90]. For time points beyond the 3–4 h post-exercise period and/or on rested days, a target meal protein intake of 0.3 g/kg would also be sufficient [17].

**Fig. 4:**
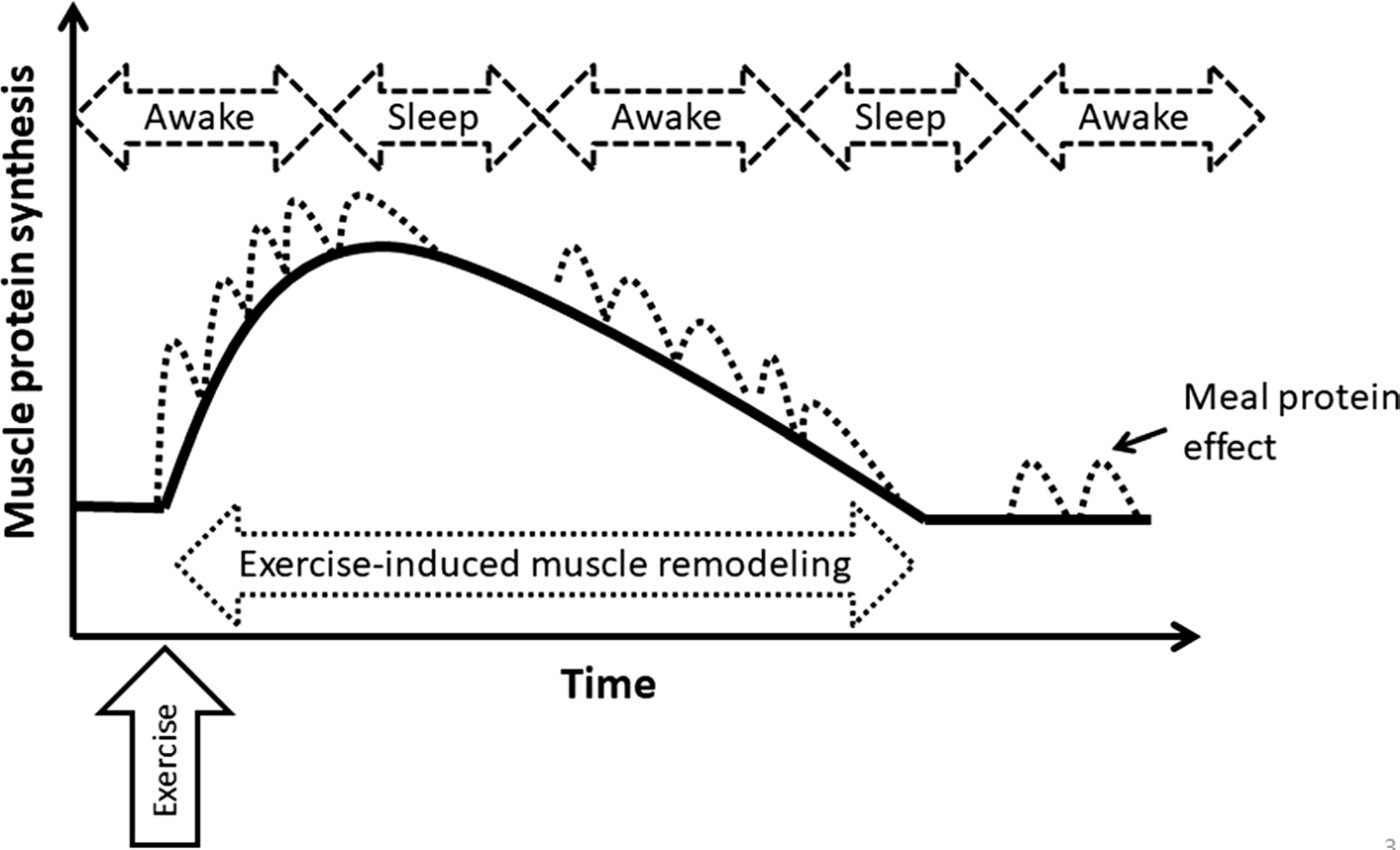
Schematic representation of the meal protein distribution pattern to optimize muscle protein synthesis after exercise (running caricature) in Master athletes. Solid line represents fasted muscle protein synthesis. Dashed lines represent an optimal meal protein-induced enhancement of muscle protein synthesis. Active caricature represents an exercise stimulus

With the goal of maximizing muscle mass and quality as a priority of most strength athletes, nutrition practices for these individuals would ideally support enhanced tissue remodeling (i.e. breakdown and synthesis of muscle protein) and a positive net protein balance. Thus, the repeated consumption of mixed macronutrient meals with an adequate quantity of protein to support MyoPS and carbohydrate to support training demands would be the cornerstone of their nutrition plan, which generally mirrors the practices of many younger elite athletes who consume 4–5 meals per day [91]. Incidentally, consuming four moderate (i.e. ~ 0.25 g/kg) whey protein doses supports greater rates of MyoPS [92] and whole body anabolism [93] than the same quantity of protein as two large (i.e. ~ 0.50 g/kg) or eight small (i.e. ~ 0.13 g/kg) meals during a prolonged 12 h post-exercise recovery period in young trained males. Moreover, a balanced distribution of protein intake as compared to a skewed intake has been reported to support moderately greater gains in lean body mass during resistance training in younger adults [94]. Thus, general recommendations for the repeated ingestion of moderate protein-containing meals every ~ 4 h (~ 4 to 5 meals) have been suggested to optimize muscle mass in trained populations [90, 95] (Fig. 4). This recommendation would likely also extend to Master athletes given the emerging evidence that meeting optimal meal protein targets is generally associated with greater lean mass and strength in their older untrained peers [96, 97], although additional research is still warranted in this field [98, 99]. However, 4–5 protein-containing meals at ~ 0.3 to 0.37 g/kg (designed to maximize muscle protein remodeling) could be manipulated to provide a daily intake (i.e. ~ 1.5 to 1.6 g/kg/day) that would be close to what has been reported to support maximal post-exercise rates of whole body protein remodeling and net protein balance [100, 101] as well as training-induced gains in fat free mass [102]. While lean body or muscle mass does not necessarily translate to greater muscle strength, it is notable that even small differences in daily protein intake (i.e. ~ 1.21 vs. ~ 1.34 g/kg/day) may be associated with detectable differences in muscle quality (total strength normalized to fat-free mass) in Master athletes [103]. It should be noted, however, that studies that specifically investigate daily protein targets typically do not provide the daily intake in a pattern that acute, lab-based studies would suggest would support an efficient muscle protein remodeling (i.e. maximal muscle protein synthesis with minimal amino acid oxidation), and therefore, might result in a slightly greater estimate. Therefore, a meal-protein approach to the dietary protein target has been advocated previously [78, 90, 104] and may be integrated more easily into most athletes’ optimal habitual meal frequency pattern (i.e. ~ 5 meals/eating occasions per day) [91] but generally skewed daily intake (i.e. the majority in the evening) [105].

## Endurance Exercise Protein Requirements

Endurance training has consistently been shown to increase daily protein requirements due in part to the requirement to replenish exercise-induced amino acid oxidative losses [1, 106]. Although there are discrepancies between methodologies used to estimate requirements in athletes, safe intakes (i.e. 2 × standard deviation or upper 95% confidence interval of the estimated average requirement) during training are ~ 50% greater than the general recommended dietary allowance by nitrogen balance (i.e. ~ 1.2 to 1.4 g/kg/day) [1, 107] and tracer-derived indicator amino acid oxidation (i.e. ~ 1.7 to 1.8 g/kg/day) [108, 109]. Incidentally, seminal research using nitrogen balance that included both younger (~ 27 years) and middle aged (~ 52 years) athletes reported identical requirements of ~ 1.2 g/kg/day [107], suggesting protein requirements in younger Master athletes are not substantially different from athletes half their age. However, the relevance of merely maintaining nitrogen balance for athletes aiming to optimize training, recovery, and/or performance has been questioned previously [110, 111]. In support, young endurance athletes who consume 1.2 g/kg/day protein and adequate carbohydrate are able to maintain protein balance during 4 days of higher volume training despite experiencing apparent losses of muscle strength and 5 km time-trial performance [112]. In contrast, when these athletes consumed 1.75 g/kg/day, which was established to be sufficient to maximize whole body protein synthesis during recovery [108], whole body net protein balance remained positive during training and was concomitant with small-to-moderate benefits (relative to 1.2 g/kg/day) in muscle strength and time trial performance [112]. Collectively, these highlight that protein requirements are towards the upper range of the current sports nutrition guidelines [3]. An intake of ~ 1.7 to 1.8 g/kg/day, which represents a modest ~ 15% of total energy, is generally met in younger athletes provided they consume adequate energy [105] but may not be reached in some Master athletes [103, 113].

Due to different methodological approaches and inherent assumptions of nitrogen balance and stable isotope tracer methodology, it may be an academic debate as to which represents the ‘true’ requirement for these endurance athletes. Thus, a meal protein intake that maximizes muscle protein remodeling may also be explored for how to parse athlete protein recommendations. Recently, Churchward-Venne et al. [114] performed an elegant study examining the protein dose–response for muscle and whole body protein synthesis after endurance exercise in trained young adults and revealed that myofibrillar, but not mitochondrial, protein synthesis was nutritionally responsive and was maximized with the equivalent of ~ 0.5 g/kg of milk protein. Whole body protein synthesis did not plateau over the range of meal protein intakes studied (i.e. up to ~ 0.65 g/kg), which confirms the greater capacity to assimilate dietary amino acids into non-muscle protein pools [22, 115] that may nevertheless still be important for an endurance athlete’s recovery (e.g. plasma volume expansion, red blood cell remodeling, bone collagen turnover, etc.) [116]. Interestingly, the relative difference (~ 0.2 g/kg) between this post-endurance exercise maximal effective dose and the previously estimated maximal dose for resistance exercise [78] is similar to the protein equivalent of what could be mobilized from exercise-induced muscle catabolism [117] and subsequently oxidized [31]. As amino acid oxidative losses (especially of the branched chain amino acids including leucine) have been suggested to underpin the greater acute [31] and daily [1, 106] protein requirements of endurance athletes, it is possible that any factor that minimizes amino acid oxidation during exercise could have the reciprocal effect of a relative reduction in the acute post-exercise protein requirement. For example, estrogen has been shown to have a protein-sparing effect with endurance exercise due to a greater ability to mobilize and oxidize fatty acids [118, 119]. Middle aged (~ 52 years) [120] and older (~ 65 years) [121] trained adults have been reported to have a greater contribution of fat to total energy expenditure during intensity-matched exercise than younger athletes. Thus, inasmuch as this greater relative fat oxidation would also be accompanied by a reduction in amino acid oxidative losses, this would further suggest Master athletes would be unlikely to have requirements that are greater than younger athletes.

From a practical standpoint, it has been suggested that protein requirements may be greater in Master athletes after strenuous bouts of exercise that may elicit some degree of acute muscle damage given the reported delay in the restoration of muscle function relative to younger athletes [122]. This recommendation is based on some of the most relevant research on Master athlete protein intakes and muscle remodeling, which demonstrated that free-living rates of MyoPS in older athletes was ~ 16% of their younger athletic peers during 3 days of recovery from a bout of downhill running despite both groups consuming an equivalent 1.6–1.7 g/kg/day protein [123]. In a follow-up study, Master athletes completed a similar bout of damaging exercise (i.e. 30 min downhill running) in the morning before consuming protein supplements (3 × 0.3 g/kg or 3 × 0.6 g/kg every 2 h) during acute recovery prior to assessing performance through a battery of tests in the same afternoon [124]. This research revealed that Master athletes consuming the high-protein intake, which would exceed current recommendations in young athletes after exercise [78] and older untrained adults at rest [17] to maximize MyoPS, had a slightly attenuated decrease in muscle strength (~ 3.6 vs ~ 8.6%, respectively) compared to the lower protein condition [124]. While this research is of potential interest to Master athletes who might train multiple times per day, it may represent a practically challenging dietary approach to adhere to given the satiating effect of this macronutrient and the requirement to achieve appropriate carbohydrate targets for optimal performance. Nevertheless, these data could align somewhat with cross-sectional research suggesting that higher, but still suboptimal (i.e. ~ 1.35 g/kg/day), daily intakes are generally associated with greater muscle function in a small cohort of mixed discipline Master athletes [103]. However, it should be noted that optimizing rates of MyoPS does not always align with enhanced recovery of muscle function [125, 126], highlighting the need for additional research that may promote the restoration of exercise performance and/or optimize training adaptations outside of what is currently recommended for muscle protein remodeling [127].

## Contemporary Sports Nutrition

### Pre-sleep Protein Ingestion

Identifying additional feeding opportunities within an athlete’s daily training schedule can help meet their generally high energy and protein demands. Young athletes have been reported to regularly have greater than four daily eating occasions (including both meals and snacks) [91, 105]. Of recent interest in augmenting muscle protein remodeling is the dietary strategy of consuming a source of protein/dietary amino acids immediately prior to sleep, which would provide amino acid precursors for protein synthesis during a period that is otherwise characterized by low circulating levels [128]. Past research in this area has generally utilized slower digesting micellar casein proteins that provide a sustained nocturnal aminoacidemia concomitant with greater rates of muscle protein synthesis [129, 130], representing a strategy that could help athletes “recover while they sleep”. Indeed, exercise in the evening before pre-sleep protein ingestion has been shown to improve overnight rates of muscle protein synthesis in older untrained adults [131]. While this dietary strategy has been shown to improve adaptations (strength and muscle hypertrophy) to resistance training in young adults [132], similar benefits have not been observed in older adults [133]. Nevertheless, the ability to enhance rates of muscle protein remodeling during an otherwise prolonged fasted period would ostensibly be a benefit (or at least not ergolytic) for Master athletes who are concurrently training. Preliminary research in young individuals suggest this could improve next day exercise performance [134] and aerobic capacity with short-term high-intensity training [135], although additional research is needed to confirm these findings and their relevance to Master athletes.

### Low Carbohydrate Availability Training

Contemporary sports nutrition approaches include the periodized intake of carbohydrate with the aim of providing just the essential fuel required for high intensity training (i.e. “fuel for the work”) and/or purposely training with low carbohydrate availability (e.g. fasted training, low daily or periodized carbohydrate intake, etc.) as a means to enhance the activation of mitochondrial biogenesis pathways, increase fat oxidation, and/or improve aerobic capacity [136]. These approaches, whether intentional or due to dietary deficiencies, are a feature of many elite endurance athlete training programs [137]. However, training with low carbohydrate availability has also been shown to augment amino acid oxidative losses as a compensatory energy source [2] that can increase daily protein requirements by ~ 10 to 15% [138], which Master athletes need to be mindful of if they incorporate this type of training into their plans.

### Intermittent Fasting

Time-restricted feeding (TRF; e.g. 16:8 h fasted/feeding) is gaining attention for the potential to increase fat oxidation via a reduced daily insulin response (e.g. 6–8 h eating window vs. the traditional 12–16 h) and alter body composition via reduced total daily energy intake and/or greater lipolysis [139]. In the event that exercise is performed early in the fasted window then the prolonged post-exercise carbohydrate restriction could align with the “recover low” principle of training [140], which is thought to further enhance metabolic adaptations and mitochondrial biogenesis. However, this condensed feeding approach may be counterproductive for protein intake patterns that would optimize skeletal muscle protein remodeling (as discussed in more detail here [141]), which currently recommends a balanced distribution of moderate (~ 0.3 g/kg) discrete protein containing meals every 3–4 h [78, 90]. This feeding approach does not appear to interfere with resistance training-induced gains in fat-free mass in young individuals consuming adequate daily dietary protein [139, 142, 143], likely owing to the ability of this exercise modality to increase the anabolic sensitivity of skeletal muscle to dietary amino acids [59, 144]. However, preliminary research suggests that fat-free mass loss may be concomitant with fat loss when applying this dietary manipulation in young endurance athletes despite no apparent advantage to aerobic capacity or performance [145], although there are reports that power/weight performance variables may be improved due to reductions in body mass [146]. Given that protection from typical age-related muscle loss is equivocal in Master endurance athletes [43, 147] and performance benefits of TRF are not apparent in young athletes [145], older endurance trained athletes are cautioned about experimenting with this dietary approach in order to avoid any unintended muscle loss. Thus, it is recommended that Master athletes prioritize a balanced daily protein ingestion to maximize muscle remodeling and mass.

### Protein Type

The majority of interventional research in sports nutrition employs a supplemental protein approach due to convenience (e.g. sports drink/bar within a controlled diet) and/or control (e.g. defined amino acid profile, digestion/absorption, macronutrient profile, etc.). This has contributed to the suggestion that leucine-enriched, rapidly digested proteins should be prioritized during the immediate post-exercise period to stimulate a rapid increase in muscle protein synthesis [95], which is based primarily on research using liquid protein beverages. While much of this research has provided the foundational knowledge for current sports nutrition recommendations, it generally belies the reality that athletes consume mixed macronutrient whole foods, although most athletes do still consume supplemental protein foods [148]. Research is beginning to reveal that whole foods may contain anti-nutritional components (e.g. phytic acids, tannins, protease inhibitors that are common to plant proteins, and/or byproducts of processing such as Maillard compounds [149]) that could interfere with the digestion and absorption of dietary amino acids and/or non-protein micronutrients or bioactives that may enhance their anabolic effects (for in-depth reviews, see [150, 151]). For example, despite providing equivalent total protein, whole eggs have been shown to more effectively activate mTORC1 [62] and support MyoPS after resistance exercise in young adults than egg whites [152]. It has also been reported that mycoprotein, a sustainably produced fungal protein, supports greater rates of mixed muscle protein synthesis after resistance exercise than a leucine-matched (but not isoprotein) quantity of milk protein [153]. Therefore, Master athletes should prioritize the consumption of nutrient and protein dense foods to meet their meal and daily protein targets. Athletes consuming a primarily or exclusively plant-based diet should ensure each meal includes complementary foods to minimize any amino acid deficiencies [154] and may consider consuming a slightly greater protein intake (e.g. ~ 10%) to account for the generally lower quality of plant-based proteins.

Master athletes may be prone to connective tissue injury such as strains and sprains or joint degradation either from acute injury or overuse [155]. Recent research suggests collagen-enriched food (e.g. gelatin) or supplements may promote collagen turnover in athletes when consumed before exercise with adequate vitamin C, the latter of which is essential for enzymes responsible for collagen cross-linking (i.e. lysyl oxidase and prolyl/lysyl hydroxyprolases) [156, 157]. While other supplements have been investigated for their potential role in preventing or facilitating recovery from injuries, the reader is referred to other reviews on this topic [158]. Thus, while collagen protein, due to its primary nonessential amino acid profile and low protein score [159], does not support contractile protein remodeling in humans [160, 161], it may be paired with other higher quality proteins to augment MyoPS [162]. However, preliminary research suggests that collagen supplementation may not enhance muscle extracellular matrix remodeling with exercise in older women [161], although the large variability in muscle collagen protein synthesis and moderate estimated effect size (i.e. 0.53; 95% CI − 0.53 to 1.53) in favor of 25 g daily collagen compared to whey protein supplementation in this study suggests additional research is warranted. Regardless, collagen is also rich in the amino acid glycine, which may be conditionally essential in humans due to a suggested deficiency in the ability of the liver and kidney to synthesize sufficient amounts in humans to satisfy the metabolic demand for collagen synthesis [163]. Moreover, collagen protein supplementation of an adequate mixed protein diet has little effect on the overall dietary amino acid quality given the relatively low collagen peptide content of most Western diets [159]. Thus, given its relatively low cost and GRAS (generally regarded as safe) status, collagen protein may be a low-risk option for Master athletes to supplement their diet.

## Impact of Sex

Despite the reportedly greater relative participation rates of females than males in some Master events (e.g. marathon) [164], there is an unfortunate under-representation of female participants in sport science research [73]. Thus, much of our sports nutrition guidelines has been derived from research in males, which has forced the field to consider how the presence or absence of sex hormones may influence protein metabolism and nutritional requirements. Premenopausal female athletes are unlikely to have substantial differences in their protein requirements after resistance exercise given that muscle protein synthesis is general consistent across the menstrual phase [165] and available evidence is lacking to suggest female athletes’ response to dietary protein is different from their male counterparts [78]. Where requirements may differ slightly is with endurance training when the estrogen:progesterone ratio is high as the former has been shown to have a protein sparing effect during aerobic exercise [118, 119]. Thus, while direct evidence across the menstrual phase is lacking, protein requirements may be a conservative ~ 10 to 15% lower during the follicular as compared to luteal phase. Additional research is required to more clearly identify whether oral contraception may be a modifying influence on protein requirements [166]. Therefore, a practical approach to ensure protein needs are met by premenopausal woman across the menstrual phase could be to adhere to general guidelines derived primarily in males, as summarized above.

Menopause is generally characterized by a temporary but accelerated loss of muscle mass that is not attenuated by hormone replacement therapy [167] but can be partially offset by resistance exercise [168, 169], highlighting the importance of muscle contraction to offset ‘normal’ age-related muscular alterations. However, post-menopause has also been reported to represent a divergence in rested and postprandial muscle protein metabolism in untrained older adults (for review, see [170]). For example, while both untrained older females and males present a general anabolic resistance to exogenous amino acids at rest, females may be able to compensate for this with a greater basal rate of muscle protein synthesis [171, 172]. Studies are currently lacking that have compared both older males and females after exercise in response to differences in dietary protein ingestion, which would be more relevant to Master athletes. When evaluating the increase in MyoPS after resistance exercise with whey protein ingestion normalized to body mass performed by a single laboratory [19, 53, 161], it was equivocal whether the response in healthy older untrained females mapped on to the response in older untrained males. For example, older males consuming ~ 0.25 or ~ 0.49 g/kg bolus of whey protein demonstrated a ~ 116% and ~ 187%, respectively, increase in MyoPS after resistance exercise. Using a similar methodology and protein source, older untrained females consuming an intermediate dose of ~ 0.37 g/kg reported an intermediate (~ 144%) [161] or a lower (~ 43%) [53] increase in MyoPS compared to the males. Thus, while much research is required to more clearly determine the specific protein requirements in postmenopausal female athletes there is currently insufficient evidence to suggest they would be substantially different from the available research in males.

## Protein Intake and Bone Health

Aging is associated with a gradual loss of bone density and strength that can predispose bones to increased fracture risk. There is evidence that chronic physical activity associated with the training of many Master athletes (especially those performing strength and power training) helps maintain bone mineral density [9, 173], which is consistent with the osteogenic nature of bone loading. However, there is also evidence that a daily protein intake greater than the current RDA of 0.8 g/kg/day is associated with greater bone mineral density [174], especially when consumed with adequate calcium and potentially vitamin D intake [175]. Given the recommendations for protein discussed above would translate into intakes that would be in excess of the current RDA, Master athletes adhering to the current guidelines would meet their protein needs for maintaining bone health. Potential caveats could be to ensure Master athletes also consume adequate calcium (i.e. > 600 mg/day), vitamin D (> 800 IU/day), and alkaline salts, the latter of which would help regulate acid–base balance, a risk factor for bone loss with age [176], and can be found in a balanced diet enriched with fruits and vegetables. Readers interested in additional nutritional recommendations to maintain bone health are referred to other reviews on this topic (e.g. [177]).

## Is There Scope for Improvement in Master Athletes?

It has been suggested that performance in Master triathletes has yet to peak [178] and may be aided by an advanced understanding of training, recovery, therapeutics, and sports nutrition. However, Master athletes self-report lower post-exercise meal protein intakes than their younger counterparts [179] and may generally consume suboptimal daily intakes (i.e. < 1.31 g/kg/day) [103], although one must be mindful of the general propensity for under-reporting in a range of dietary assessment strategies [180]. These lower intakes may also reflect a lack of knowledge on the part of some athletes as 50% of older triathletes responded “I don’t know” when asked about an optimal post-exercise protein dose, whereas only 22% responded with (at the time of its publication) the recommended 20–25 g protein [179]. Importantly, in light of a recent post-endurance exercise protein dose–response study [114], younger athletes on average consumed close to the recommended intake of ~ 0.5 g/kg, whereas older adults were below this new target (i.e. ~ 0.4 vs. 0.3 g/kg, respectively) [179]. Therefore, increasing total daily protein intake and/or meal protein during the early post-exercise recovery period may represent a safe and feasible means for Master athletes to enhance their recovery, adaptation, and performance.

## Conclusion

Similar to their younger peers, Master athletes should ensure their nutrition plans include adequate dietary protein intake to support their training and recovery. Fortunately, current evidence does not support the need for higher protein intakes than what is currently recommended for and developed in younger athletes as much of the discussion on increased protein requirements in older adults to offset any age-related anabolic resistance is of little relevance to highly active Master athletes. Athletes who apply a dietary approach that targets an optimal meal protein intake and frequency to efficiently stimulate muscle protein remodeling (in addition to other important aspects of recovery such as optimal carbohydrate and energy intake) would likely reach a daily protein intake that would approximate ‘optimal’ daily protein estimates from studies that do not specifically control for meal timing or pattern (summarized in Table 1); however, it is argued that a meal protein approach, which may yield a slightly lower daily estimate due to a more efficient protein intake, would be more practical for dietary planning while also ensuring a balanced daily protein distribution for optimal muscle remodeling. While additional research could confirm whether current recommendations for Master athletes are indeed optimal, the ability of Master athletes to age gracefully and successfully allows them to look back at what supports their younger contemporaries as a strong foundation on which to build their success going forward.

**Table 1:** Recommended meal protein intakes for Master athletes

	Post-exercise^1^	Daily^2^	Considerations
Endurance training^3^	0.5 g/kg per meal Rapidly digested, leucine-enriched	0.3–0.4 g/kg per meal High quality, nutrient dense (e.g. whole foods) Enriched in branched chain amino acids Consume 4–5 equally spaced meals	Target a daily intake of ~ 1.8 g/kg/day with adequate energy^5^ Include ~ 10% buffer with lower quality proteins (e.g. plant-based) Requirements may be increased ~ 10 to 15% with low carbohydrate availability training If tolerable, target last meal ~ 1 to 2 h before sleep
Resistance training^4^	0.3–0.4 g/kg per meal Rapidly digested, leucine-enriched	0.3–0.4 g/kg per meal High quality, nutrient dense (e.g. whole foods) Consume 4–5 equally spaced meals	Target a daily intake of ~ 1.6 g/kg/day with adequate energy^5^ Include ~ 10% buffer with lower quality proteins (e.g. plant-based) If tolerable, target last meal ~ 1–2 h before sleep

^1^Post-exercise refers to the first meal after exercise (i.e. within ~ 1 h after training cessation to maximize muscle protein synthesis)

^2^Daily meals refer to all meals throughout the day with the exception of the post-exercise meal

^3^Endurance training refers to aerobic-based exercise of moderate–high intensity (e.g. ≥ 70% *V*O_2peak_)

^4^Resistance training refers to high effort, externally loaded muscle contractions (e.g. weight lifting)

^5^Daily protein targets extracted from studies that have not specifically used a summative target meal protein approach for maximizing muscle protein synthesis
